# Mutations in the promoter region of methionine transporter gene *metM* (Rv3253c) confer *para*-aminosalicylic acid (PAS) resistance in *Mycobacterium tuberculosis*

**DOI:** 10.1128/mbio.02073-23

**Published:** 2024-01-05

**Authors:** Yu Zhang, Shiyong Wang, Xinchang Chen, Peng Cui, Jiazhen Chen, Wenhong Zhang

**Affiliations:** 1 Department of Infectious Diseases, Shanghai Key Laboratory of Infectious Diseases and Biosafety Emergency Response, National Medical Center for Infectious Diseases, Huashan Hospital, Shanghai Medical College, Fudan University, Shanghai, China; 2 Shanghai Huashen Institute of Microbes and Infections, Shanghai, China; 3 National Clinical Research Center for Aging and Medicine, Huashan Hospital, Fudan University, Shanghai, China; 4 Key Laboratory of Medical Molecular Virology (MOE/MOH), Shanghai Medical College, Fudan University, Shanghai, China; The Hebrew University of Jerusalem, Rehobot, Israel

**Keywords:** *Mycobacterium tuberculosis*, resistance, *para*-aminosalicylic acid, *metM *promoter, methionine

## Abstract

Tuberculosis (TB) is a significant global public health threat. Despite the long-standing use of *para*-aminosalicylic acid (PAS) as a second-line anti-TB drug, its resistance mechanism remains unclear. In this study, we isolated 90 mutants of PAS-resistant *Mycobacterium tuberculosis* (MTB) H37Ra in 7H11 solid medium and performed whole-genome sequencing, gene overexpression, transcription level comparison and amino acid level determination in MTB, and promoter activity by β-galactosidase assays in *Mycobacterium smegmatis* to elucidate the mechanism of PAS resistance. Herein, we found that 47 of 90 (52.2%) PAS-resistant mutants had nine different mutations in the intergenic region of *metM* (*Rv3253c*) and *Rv3254*. Beta-galactosidase assays confirmed that mutations increased promoter activity only for *metM* but not *Rv3254*. Interestingly, overexpression of MetM or its *M. smegmatis* homolog (MSMEI_1796) either by its promoter in *metM*’s direction or by exogenous expression in MTB induced PAS resistance in a methionine-dependent manner. Therefore, drug susceptibility results for the *metM* promoter mutants can be misleading when using standard 7H10 or 7H9 medium, which lacks methionine. At the metabolism level, PAS treatment led to higher intracellular methionine levels in the mutants than the wild type, antagonizing PAS and conferring resistance. Furthermore, 12 different mutations in the *metM* promoter were identified in clinical MTB strains. In summary, we found a novel mechanism of PAS resistance in MTB. Mutations in the *metM* (Rv3253c) promoter upregulate *metM* transcription and elevate intracellular methionine, which antagonize PAS. Our findings shed new light on the mechanism of PAS resistance in MTB and highlight issues with the current PAS susceptibility culture medium.

**IMPORTANCE:**

Although *para*-aminosalicylic acid (PAS) has been used to treat TB for more than 70 years, the understanding of PAS resistance mechanisms is still vague, living gaps in our ability to predict resistance and apply PAS effectively in clinical practice. This study aimed to address this knowledge gap by inducing *in vitro* PAS resistance in *Mycobacterium tuberculosis* (MTB) using 7H11 medium and discovering a new PAS resistance mechanism. Our research revealed that spontaneous mutations occurring in the promoter region of the methionine transporting gene, *metM*, can upregulate the expression of *metM*, resulting in increased intracellular transport of methionine and consequently high-level resistance of *Mycobacterium tuberculosis* to PAS. Notably, this resistance phenotype cannot be observed when using the commonly recommended 7H10 medium, possibly due to the lack of additional methionine supply compared with that when using the 7H11 medium. Mutations on the regulatory region of *metM* were also found in some clinical MTB strains. These findings may have important implications for the unexplained PAS resistance observed in clinical settings and provide insight into the failures of PAS treatment. Additionally, they underscore the importance of considering the choice of culture media when conducting drug susceptibility testing for MTB.

## INTRODUCTION

Tuberculosis (TB) remains a formidable threat to global public health and is the leading cause of death from a single infectious agent prior to the onset of the coronavirus disease 2019 (COVID-19) pandemic, surpassing even HIV/AIDS in its lethality (1). In 2020 alone, an estimated 9.9 million new TB cases were reported, equivalent to 127 cases per 100,000 population (1). The prevalence of multidrug-resistant TB (MDR-TB) or rifampin-resistant TB stands at approximately 3%–4% among newly diagnosed patients and 18%–21% among patients with prior treatment history (1). *Para*-aminosalicylic acid (PAS), initially employed in TB treatment in 1946 (2), has emerged as a second-line anti-TB drug. Given the rise of MDR-TB, PAS is frequently included in MDR-TB regimens as a Group C drug during the intensive phase of treatment (3).

While the application of PAS in combination with streptomycin to reduce the emergence of drug-resistant TB dates back to 1952 (4), the precise mechanism of resistance remains elusive. As a prodrug activated through the folate synthesis pathway (5), PAS can be bioconverted into folate intermediate analogs, hydroxy-H_2_Pte and hydroxy-H_2_PteGlu, thereby disrupting folate metabolism of *Mycobacterium tuberculosis* (MTB) (5–7).

Known PAS resistance-associated genes, including *thyA*, *dfrA*, *folC*, and *ribD*, encode enzymes responsible for folate biosynthesis in MTB (7–10). In clinical isolates from China, mutations in *folC* are the most frequently encountered and found in PAS-resistant clinical isolates (10–12). Research involving spontaneous PAS-resistant mutant strains of MTB H37Rv, H37Ra, and *M. bovis* bacille Calmette-Guérin (BCG) has identified *folC* mutations as conferring PAS resistance (7, 9). In contrast, mutations in *thyA*, *dfrA*, and *ribD* were initially discovered using a transposon mutant library in *M. bovis* BCG (8). Mutations in *thyA*, responsible for encoding a folate-dependent thymidylate synthase, were first identified in PAS-resistant *M. bovis* BCG transposon mutants and subsequently found in clinical isolates exhibiting up to 100-fold PAS resistance (8, 13). Overexpression of *dfrA* or *ribD* also led to PAS resistance in MTB. At high expression levels, RibD can function as an alternative dihydrofolate reductase (DHFR), the target of PAS, compensating for a genetic deletion of *dfrA* which encodes DHFR in MTB. A spontaneous mutation located 11 bp upstream of *ribD* in an MTB isolate increased RibD expression, resulting in PAS resistance (7).

The occurrence of clinical PAS-resistant strains with mutations in the aforementioned genes ranged from 61.1% to 91.8% (10–12). While these results are not entirely consistent, they collectively suggest that known resistant mutations cannot account for all PAS resistance cases. Previous studies have sought to elucidate other unknown resistant mechanisms via *in vitro* screening of MTB mutants in PAS-containing medium (14). Multi-omics comparisons between *folC*-mutant and non-*folC* PAS-resistant strains revealed that non-*folC* mutants exhibited increased uptake of exogenous methionine, mitigating the effects of inhibitors and enhancing DfrA and ThyA expression.

In this study, we performed an *in vitro* experiment to induce PAS resistance in MTB using 7H11 medium and identified novel mutations conferring PAS resistance. Through whole-genome sequencing (WGS), quantitative real-time PCR (RT-qPCR), β-galactosidase assays, and gene overexpression experiments, we uncovered a new mechanism of PAS resistance in 7H11 medium associated with mutations in the promoter region of *metM* encoding a methionine transporter that will most likely go unnoticed when using 7H10 solid medium deficient in methionine, thereby shedding new light on the mechanism of PAS resistance in MTB.

## RESULTS

### PAS-resistant MTB mutants carried *metM* promoter region mutations

To uncover potential novel loci associated with PAS resistance, approximately 1 × 10^8^ CFUs of MTB H37Ra were plated on 7H11 plates supplemented with 10% Albumin Dextrose Catalase (ADC) and varying PAS concentrations (4 or 16 µg/mL). Following 4–6 weeks of incubation, 90 mutants grew and were picked from PAS-containing plates. Subsequent DNA sequencing revealed that only four mutants exhibited *folC* mutations, specifically g145a, t458c, t458g, and a128c. Among the 90 mutants, a substantial 47 (52.2%) isolates carried mutations in the intergenic region between *metM* (*Rv3253c*, encoding a putative amino acid transporter) and *Rv3254* (encoding an uncharacterized protein). Out of these 47 isolates, 34 harbored a single mutation identified by WGS, while mutations in 11 isolates were identified via Sanger sequencing (Table 1). Besides the intergenic region of *metM* and *Rv3254*, two isolates presented mutations in *glmS* (*Rv3436c*, encoding glutamine-fructose-6-phosphate aminotransferase) and *Rv1363c* (encoding an uncharacterized protein), respectively. In total, nine mutations (G-7T, G-31A, G-33A, G-35T, A-39G, C-40T, C-42T, C-57T, and C-101T) were detected in the *metM* non-coding region (Table 1).

**TABLE 1:** The intergenic region mutations identified in PAS-resistant MTB mutants

Strains	FolC	Base changes (according to *metM*)	Base changes (according to *Rv3254*)	Other mutations
P32	WT	C−7T	G−84A	No
P114	WT	C−7T	G−84A	No
P8	WT	G−31A	C−60T	No
P9	WT	G−31A	C−60T	No
P10	WT	G−31A	C−60T	No
P11	WT	G−31A	C−60T	No
P12	WT	G−31A	C−60T	No
P13	WT	G−31A	C−60T	No
P14	WT	G−31A	C−60T	No
P15	WT	G−31A	C−60T	No
P18	WT	G−31A	C−60T	No
P19	WT	G−31A	C−60T	*Rv3436c* (a1658t, Q553L)
P20	WT	G−31A	C−60T	No
P21	WT	G−31A	C−60T	No
P22	WT	G−31A	C−60T	No
P23	WT	G−31A	C−60T	No
P25	WT	G−31A	C−60T	No
P26	WT	G−31A	C−60T	No
P27	WT	G−31A	C−60T	No
P28	WT	G−31A	C−60T	No
P29	WT	G−31A	C−60T	No
P75	WT	G−31A	C−60T	No
P76	WT	G−31A	C−60T	No
P80	WT	G−31A	C−60T	No
P139	WT	G−31A	C−60T	No
P30	WT	G−33A	C−58T	No
P41	WT	G−33A	C−58T	No
P62	WT	G−33A	C−58T	WGS not conducted
P91	WT	G−33A	C−58T	No
P113	WT	G−33A	C−58T	No
P79	WT	C−35T	G−56A	WGS not conducted
P81	WT	C−35T	G−56A	WGS not conducted
P94	WT	C−35T	G−56A	WGS not conducted
P120	WT	A−39G	T−52C	WGS not conducted
P97	WT	C−40T	G−51A	WGS not conducted
P24	WT	C−42T	G−49A	WGS not conducted
P61	WT	C−42T	G−49A	WGS not conducted
P89	WT	C−42T	G−49A	WGS not conducted
P90	WT	C−42T	G−49A	WGS not conducted
P39	WT	C−57T	G−34A	No
P55	WT	C−57T	G−34A	No
P70	WT	C−57T	G−34A	No
P71	WT	C−57T	G−34A	WGS not conducted
P31	WT	C−101T	G11A	No
P34	WT	C−101T	G11A	No
P50	WT	C−101T	G11A	No
P122	WT	C−101T	G11A	*Rv1363c* (a400g, T134A)

**Fig 1:**
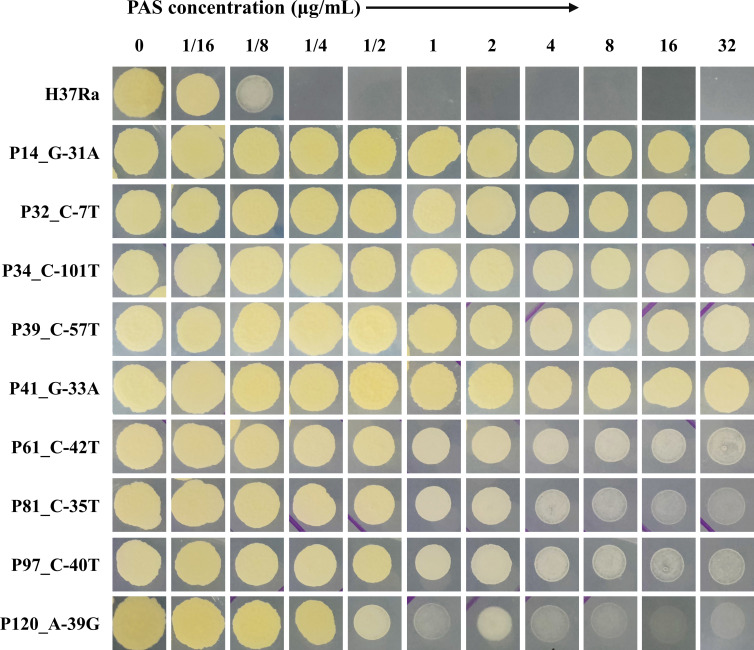
Mutant strains were resistant to PAS compared with the wild-type strain H37Ra. Each strain was inoculated at a density of approximately 3 × 10^7^ CFU/mL.

PAS susceptibility tests were performed for all mutants and the wild-type H37Ra strain on 7H11 agar plates. The results demonstrated that all nine mutants exhibited heightened resistance to PAS. Notably, mutants P61, P81, P97, and P120 displayed clear advantages over the wild-type strain at a PAS concentration of 32 µg/mL. The growth rates of mutants P14, P32, P34, P39, and P41 even surpassed those of the former four strains at the same PAS concentration (Fig. 1).

**Fig 2:**
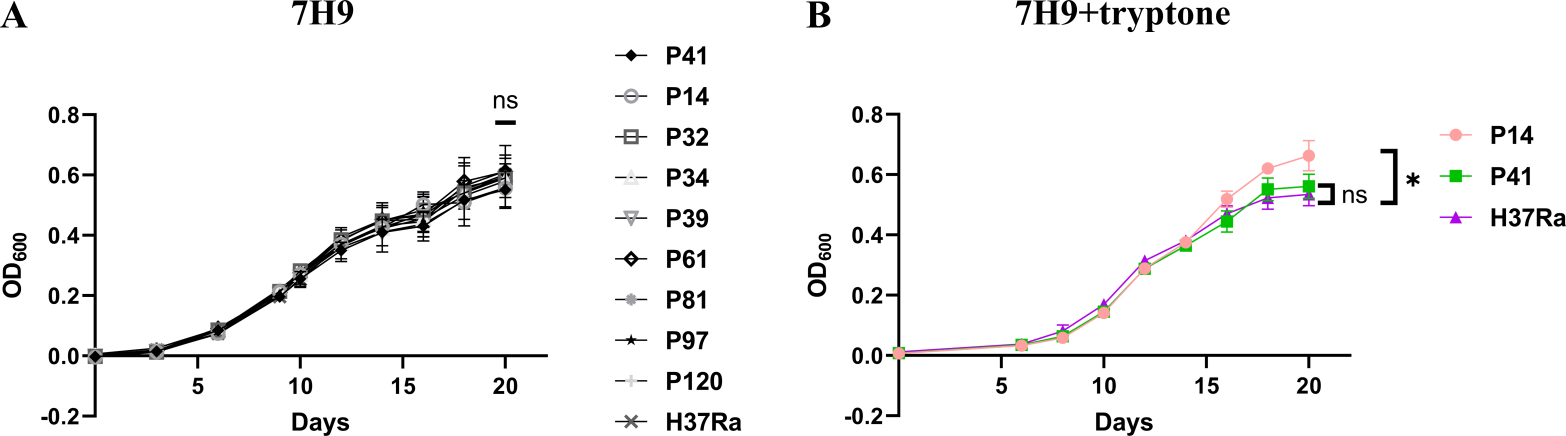
Growth curves. (**A**) Growth of MTB H37Ra and each mutant strain in 7H9 broth supplemented with 10% ADC. (**B**) Growth of H37Ra, P14, and P41 in 7H9 supplemented with 10% ADC and 1 g/L tryptone. The average of three biological replicates is shown. ns, not significant. *P < 0.05.

The growth curve revealed no significant growth defects among the mutant strains compared with the wild-type strain in 7H9^ADC^ broth (Fig. 2A). In fact, mutant P14 displayed a slight growth advantage on day 20 when supplemented with 1 g/L of tryptone (Fig. 2B).

### Relative expression levels of *metM* and *Rv3254* and β-galactosidase assays for promoter activity determination

**Fig 3:**
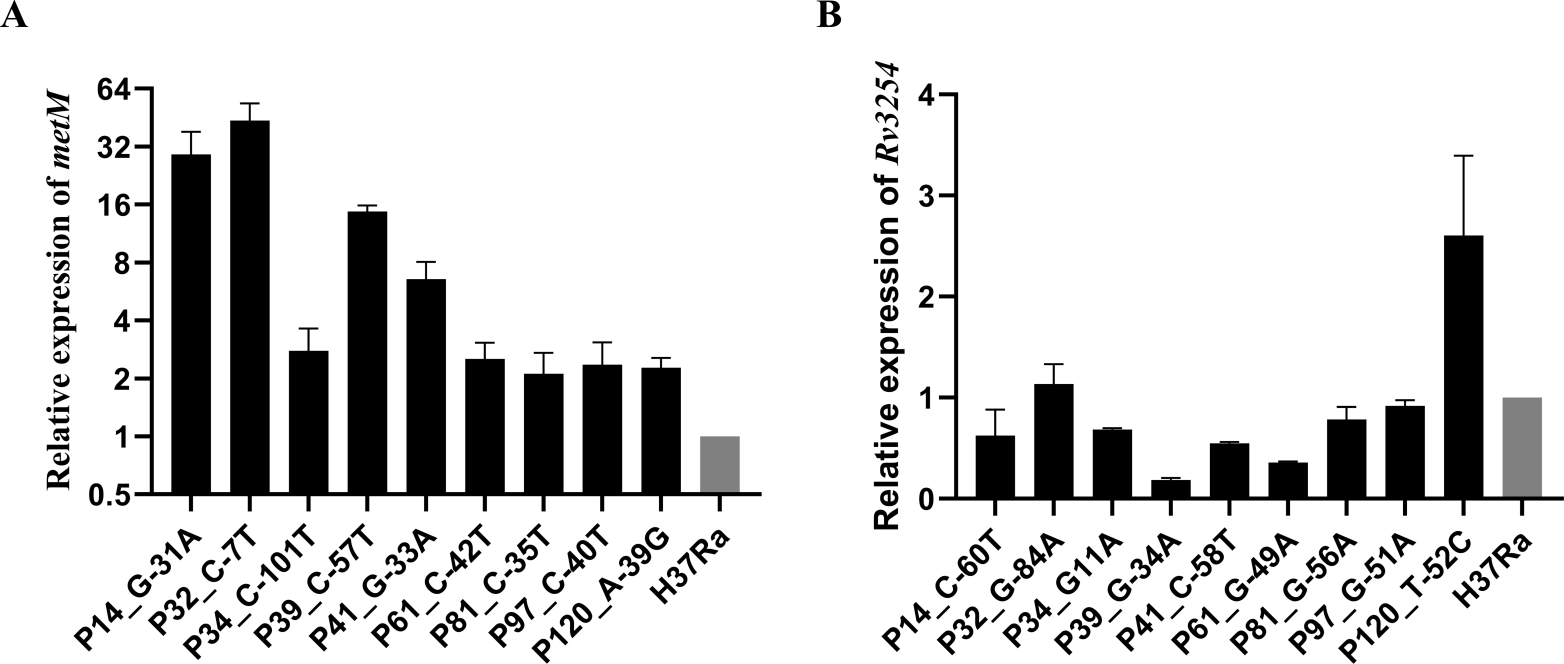
The relative expression of *metM* and *Rv3254*. (**A**) The relative expression of *metM* in each mutant strain. Four biological replicates were performed. (**B**) The relative expression of *Rv3254* in each mutant strain. Two biological replicates were performed. Ra represents the wild-type strain H37Ra.

Given that the mutations were found in the 5′-end non-coding regions of both *metM* and *Rv3254*, it was crucial to determine which gene exhibited changes in expression. The expression levels of *metM* in all mutants displayed a significant increase, ranging from 2.1-fold (P81) to 43.7-fold (P32) compared with those in H37Ra as revealed by RT-PCR. However, the expression levels of *Rv3254* did not significantly differ between the mutant strains and the wild-type strain (Fig. 3A and B).

**Fig 4:**
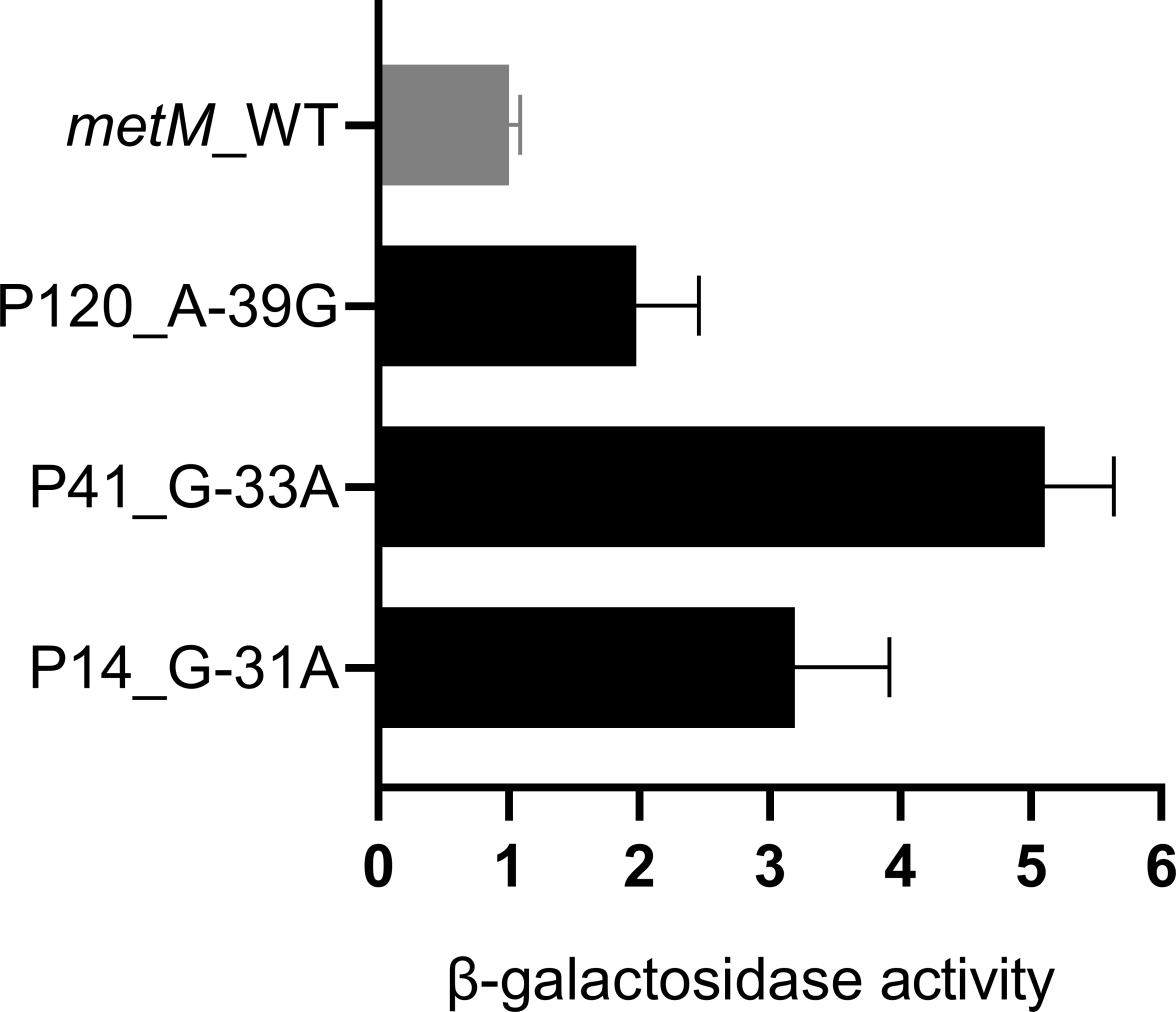
β-Galactosidase activity of *metM* promoter mutant and wild-type promoters. The promoter activity was represented by β-galactosidase activity on promoter-lacZ recombinant plasmids in *M. smegmatis. metM* promoter single-nucleotide polymorphisms elevated the expression of the downstream gene *lacZ* compared with the findings for the wild type. Three biological replicates were performed. *P*-value: A-39G/WT, 0.0066; G-33A/WT, 0.0002; and G-31A/WT, 0.0259.

To validate the impact of mutations on promoter activity, the activities of the *metM* and *Rv3254* mutant promoters were quantified using β-galactosidase assays. For *metM*, the activities of all three mutant promoters, namely, P14 (A-39G), P41 (G-33A), and P120 (G-31A), were significantly higher (2.0–5.1-fold) than that of the wild-type promoter (*P* < 0.05, Fig. 4). On the contrary, when we assessed the promoter activity of *Rv3254*, it was not linearly correlated with optical density at 420 nm (OD_420_) at 30 min (Fig S1). Consequently, the results verified that mutations in the intergenic region led to increased promoter activity solely for *metM*.

### Overexpression of *metM* and its homolog *MSmetM* under homogeneous or heterogeneous promoters conferred PAS resistance to MTB

To substantiate the impact of elevated *metM* expression on PAS resistance, four different *metM*-overexpressing strains based on H37Ra were constructed. *metM* overexpression in the presence of either the wild-type or mutant promoter (C-57T), as well as overexpression of *M. smegmatis* or *M. tuberculosis metM* under the *lysG* promoter, was successful. Relative to the findings for the wild-type strain, the relative expression of *metM* was 40.5 ± 3.8-, 101.6 ± 10.3-, and 205.1 ± 28.5-fold higher in Ra::*metM*OE, Ra::C-57Tpro-*metM*, and Ra::*lysG*pro-*metM*, respectively. Positive detection of *MSmetM* in Ra::*lysG*pro-*MSmetM* confirmed its successful ectopic expression.

**Fig 5:**
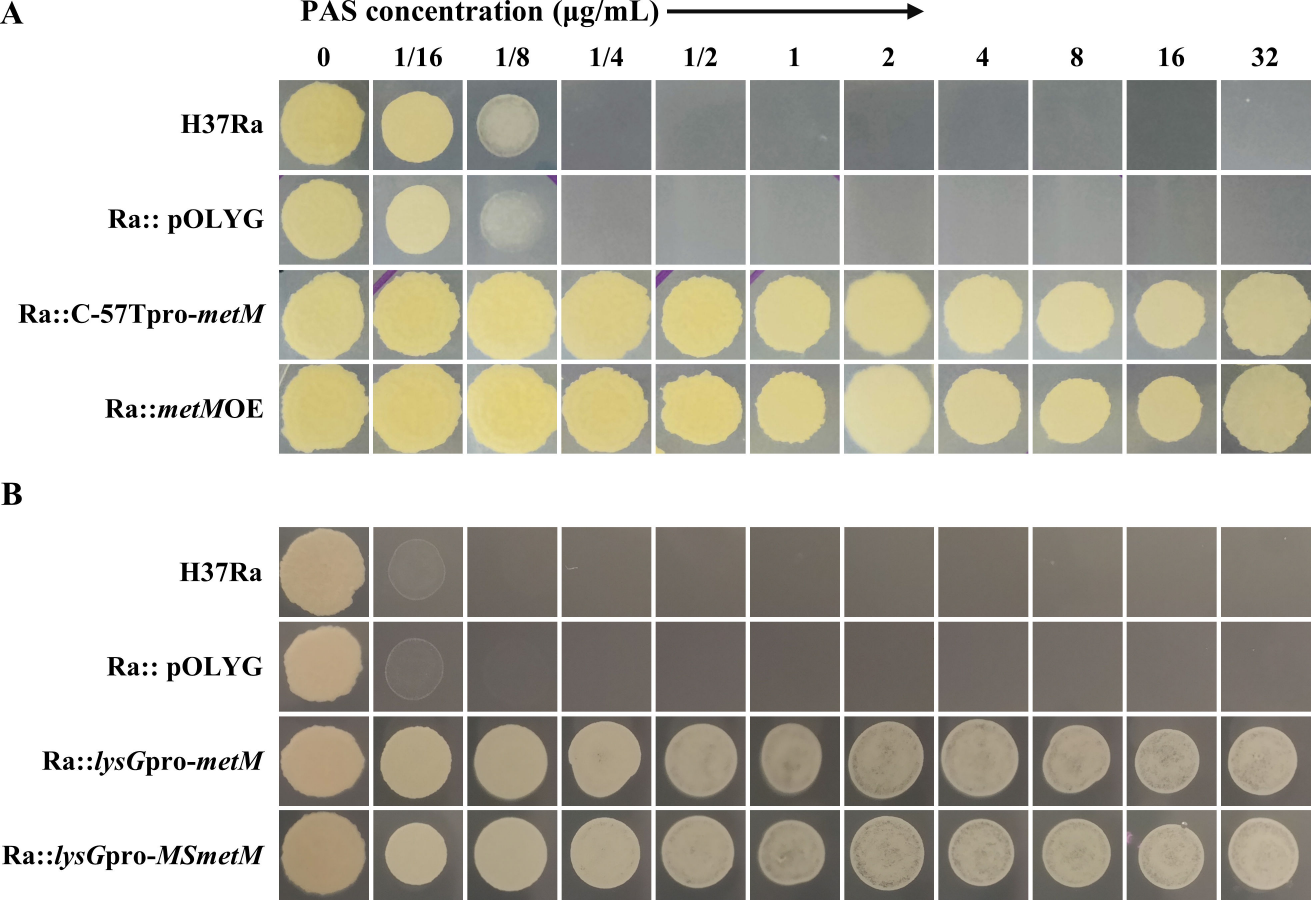
PAS susceptibility tests of *metM*-overexpressing strains. All *metM*-overexpressing strains under either wild-type or mutant *metM* promoter (**A**) and heterogeneous promoter (*lysG* promoter) (**B**) were significantly resistant to PAS compared with the wild-type strain H37Ra in 7H11 medium. Each strain was inoculated with two replicates at a density of approximately 3 × 10^7^ CFU/mL. The vector control Ra::pOLYG remained susceptible to PAS. The reference strain H37Ra in Fig. 5A was the same as that in Fig. 1.

PAS susceptibility testing on 7H11^ADC^ plates demonstrated that all strains overexpressing MTB *metM* or *M. smegmatis metM* exhibited significant PAS resistance, whereas the strain carrying the control vector remained susceptible (Fig. 5). Collectively, the results affirmed that *metM* promoter mutations induced PAS resistance by elevating *metM* transcription, rather than altering the functionality of the promoter itself. Furthermore, both *metM* in *M. smegmatis* and MTB similarly conferred PAS resistance to MTB.

### Methionine-dependent PAS resistance of mutant MTB strains and *metM*-overexpressing MTB strains

**Fig 6:**
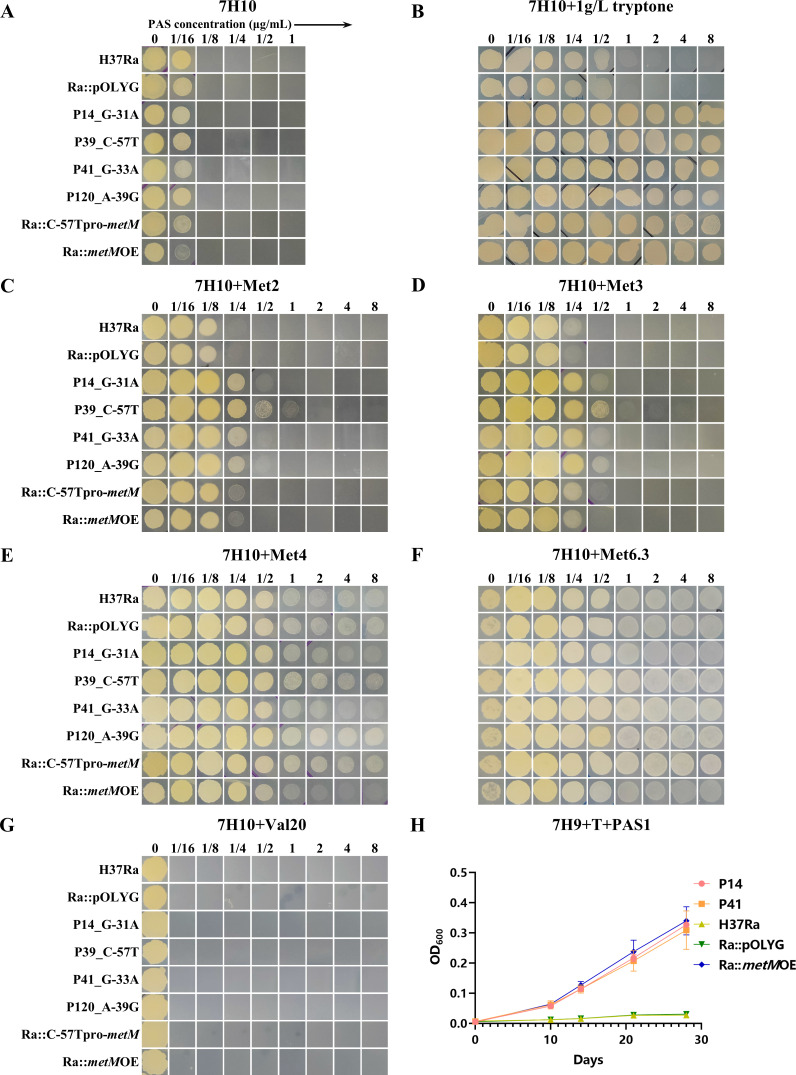
Effect of the methionine concentration on PAS susceptibility. The mutant strains P14, P39, and P41; wild-type strain H37Ra, and overexpression strains Ra::C-57Tpro-*metM* and ra::*metM*OE on (**A**) 7H10^ADC^ agar without amino acid supplementation. (**B**) 7H10^ADC^ agar supplemented with 1 g/L tryptone. (**C**) 7H10^ADC^ agar supplemented with 2 µg/mL methionine. (**D**) 7H10^ADC^ agar supplemented with 3 µg/mL methionine. (**E**) 7H10^ADC^ agar supplemented with 4 µg/mL methionine. (**F**) 7H10^ADC^ agar supplemented with 6.3 µg/ml methionine. (**G**) 7H10^ADC^ agar supplemented with 20 µg/mL valine. (**H**) 7H9^ADC^ broth supplemented with 1 g/L tryptone.

Intriguingly, on 7H10 agar plates, neither the mutants nor the *metM*-overexpressing strains displayed PAS resistance (Fig. 6A). After several repetitions on 7H10^ADC^ agar, we scrutinized the ingredients of 7H11 and 7H10 media in an attempt to discern the cause of differences in the PAS resistance phenotype. 7H11, but not 7H10, contains 1 g/L pancreatic digest of casein, also known as tryptone, which provides free amino acids and nitrogen. Upon the addition of 1 g/L tryptone to 7H10 medium, all *metM* promoter mutants and overexpression strains exhibited greater resistance to PAS (Fig. 6B). Similarly, the PAS resistance phenotype was observed in 7H9 broth supplemented with 1 g/L tryptone but not in 7H9 alone, which lacks tryptone in the medium gradient (Fig. 6H). In other words, the PAS resistance of all *metM* promoter mutant strains would be missed and could potentially yield false susceptible results when using 7H10 or 7H9 medium.

Methionine, as opposed to valine or tyrosine, mediated PAS resistance (Fig. 6). Given that methionine is known to mediate PAS antagonism, we proceeded to verify the effect of amino acids, including methionine, on PAS resistance in *metM* promoter mutants and overexpression strains. When a low concentration of methionine (2–3 µg/mL) was added to the medium, the PAS resistance of all strains increased, with mutant and overexpression strains displaying two- to eightfold higher resistance than the control strain (Fig. 6C and D). At concentrations of 4 µg/mL or higher, methionine caused significant PAS antagonism, rendering all strains, including the wild-type strain H37Ra, highly resistant to PAS. As a result, no noticeable differences in susceptibility were observed among these strains (Fig. 6E and F). Conversely, valine (Fig. 6G) and tyrosine (data not shown) had no impact on PAS resistance at a concentration of 20 µg/mL. These results underscored the methionine-dependent nature of the increased PAS resistance in *metM* promoter mutants and *metM*-overexpressing strains.

### Intracellular methionine concentration was significantly elevated in mutant MTB strains and *metM*-overexpressing strains

**Fig 7:**
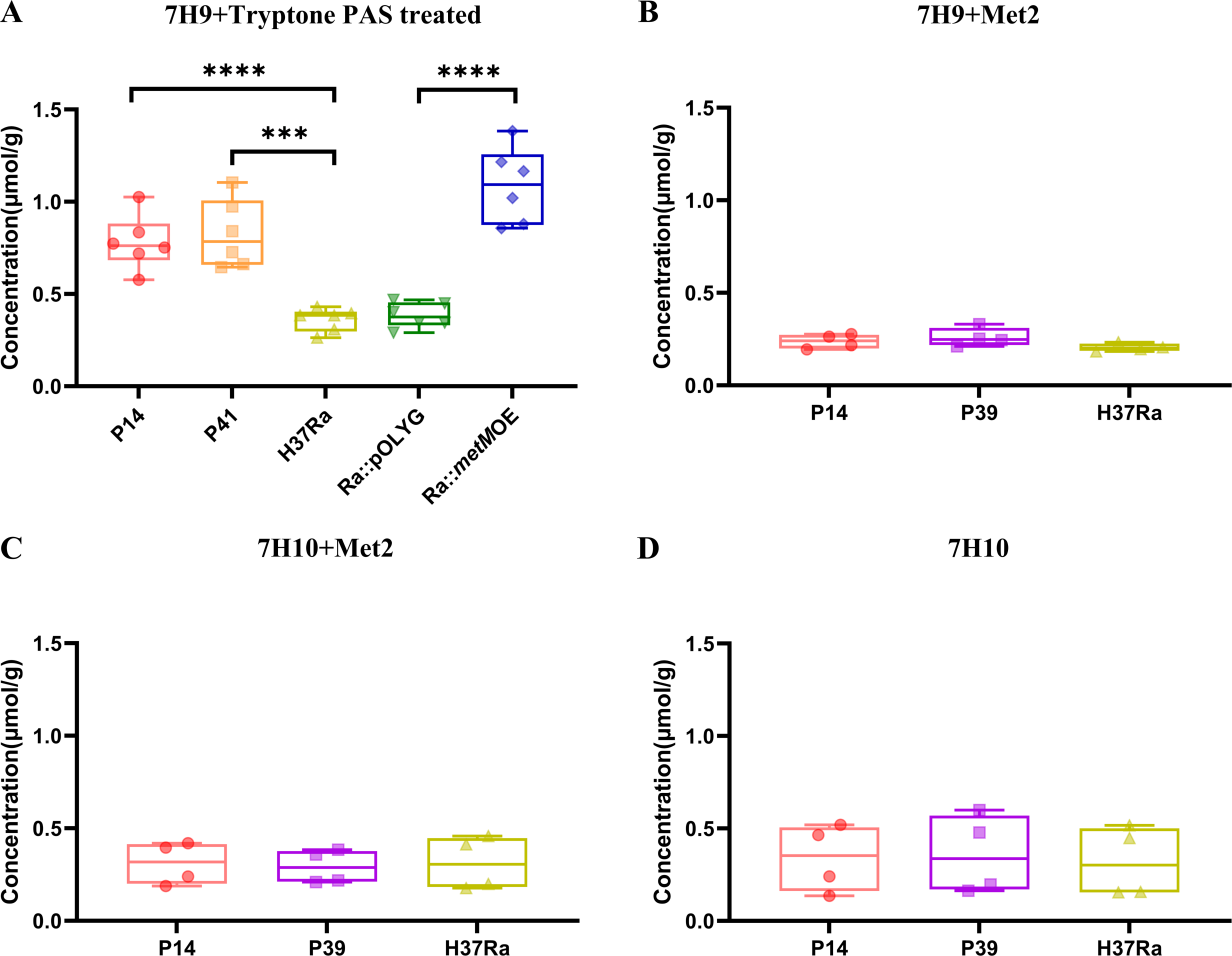
The intracellular free methionine concentration under different conditions. (**A**) Treatment with 1 µg/mL PAS for 15 h in the log phase in 7H9^ADC^ broth supplemented with 1 g/L tryptone. Growth in the absence of PAS in 7H9^ADC^ broth supplemented with 2 µg/mL methionine (**B**), on 7H10^ADC^ agar supplemented 2 µg/mL methionine (**C**), and on 7H10^ADC^ agar without methionine (**D**). ***P < 0.001 and ****P < 0.0001.

Considering that MetM is a methionine transporter in *M. bovis*, we measured the intracellular concentration of natural amino acids, including methionine, in the mutants and *metM*-overexpressing strains. Interestingly, the intracellular methionine concentrations in P14, P41, and *metM*-overexpressing strains increased by more than twofold versus that in the control strain only after treatment with PAS in the log phase in 7H9^ADC^ broth supplemented with 1 g/L tryptone (Fig. 7A). No significant increases in methionine concentration were observed when the strains were cultured in 7H9^ADC^ broth or on 7H10^ADC^ agar without PAS treatment, regardless of methionine supplementation (Fig. 7B through D). Additionally, no significant differences were noted in the concentrations of intracellular amino acids other than methionine under this condition (Table S1). These findings suggest that PAS challenge induces methionine accumulation solely in mutants with enhanced *metM* promoter activity, thereby antagonizing the effect of PAS and conferring resistance.

### *metM* promoter mutations were detected in clinical strains

**Fig 8:**
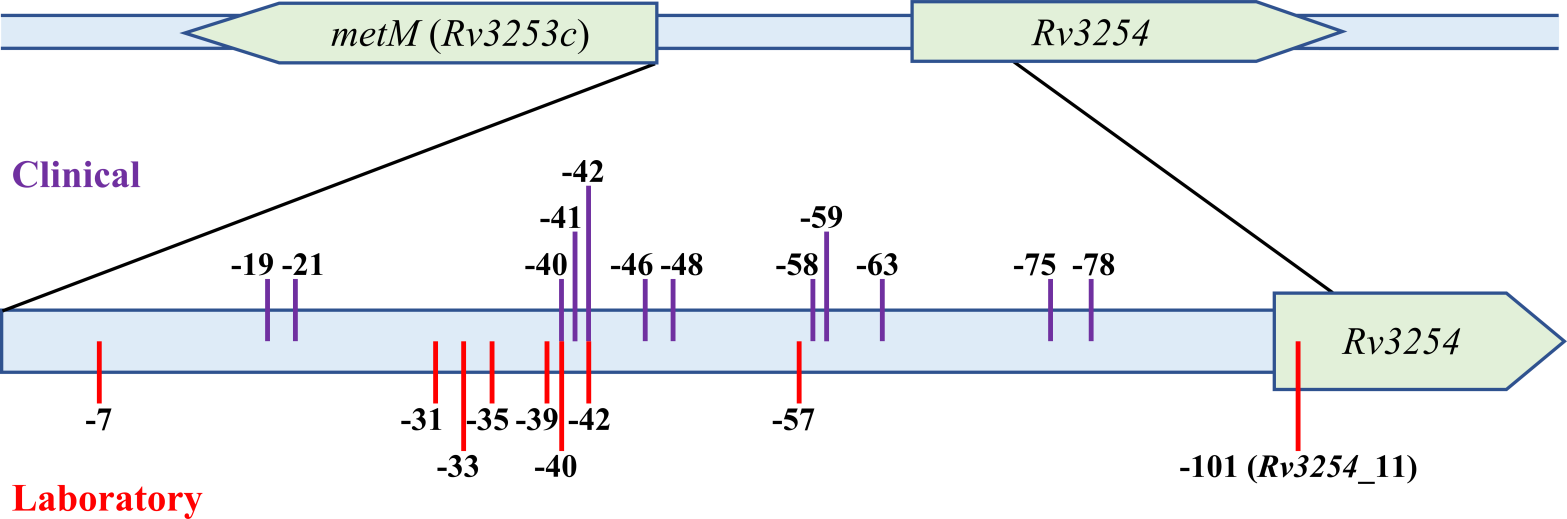
Schematic diagram of the relative locations of *metM* promoter mutations in laboratory and clinical strains. Purple lines represent mutations arising in clinical MTB strains. Red lines represent mutations arising in laboratory-induced PAS-resistant strains.

We screened for mutations in the intergenic region of *metM* and *Rv3254* in clinical MTB isolates from public databases, uncovering 12 distinct mutations (Table S2). Although we could not confirm the PAS resistance of the clinical strains in the public database, mutations at the same site (C-42T) were observed in both laboratory (P61) and clinical strains (UM200902T0099). The *metM* promoter mutants clustered in two hotspot regions, namely, −42 to −39 and −59 to −57, suggesting that these two regions could be of significance in increasing *metM* promoter activity (Fig. 8).

## DISCUSSION

The emergence of resistance to first-line anti-TB drugs in MTB has highlighted the importance of second-line agents. With the rising incidence of MDR-TB and extensively drug-resistant (XDR)-TB, PAS is often incorporated to strengthen regimens for XDR-TB patients unresponsive to MDR-TB therapy. Clinical trials have been conducted to identify the most appropriate PAS-containing regimens for patients with MDR-TB or XDR-TB (15, 16). In the current clinical research, WGS is increasingly employed in lieu of traditional susceptibility methods to diagnose second-line drug resistance in TB (17–19). However, PAS resistance based solely on WGS is challenging, as some PAS resistance mechanisms remain unidentified. In this study, we elucidated a novel PAS resistance mechanism, namely, *metM* promoter mutation, in both laboratory-generated PAS-resistant mutants and clinical MTB strains. Although prior studies have utilized *in vitro* screening of spontaneous PAS resistance experiments to identify new PAS resistance genes (9, 14, 20), these studies used Middlebrook 7H10 agar or 7H9 broth, as recommended by the Clinical and Laboratory Standards Institute (14, 21, 22). Our research demonstrates that *metM* promoter mutations bestow a unique methionine-dependent PAS resistance mechanism in MTB, which is undetectable in 7H9 or 7H10 medium, both of which lack methionine. It is worth noting that the inclusion of methionine at specific concentrations renders PAS resistance detectable, making 7H11 medium a more accurate reflection of *in vivo* methionine concentrations in human plasma (approximately 10–40 µmol/L or 1.49–5.97 µg/mL) (23) than 7H10 medium.

Bacteria resistance discrepancy between *in vivo* and *in vitro* has long been discussed and debated, especially in non-tuberculosis mycobacterium (24, 25). One prime example is the disparity between the *in vitro* and *in vivo* fosfomycin susceptibility tests involving the Uhpt membrane transporter. Since both glucose-6-phosphate (G6-P) and fosfomycin use the same transporter, Uhpt (26), which is also a G6-P-induced expression protein, partial strains showed drastically different fosfomycin MICs on G6-P synthetic and non-synthetic media (27). Therefore, for accurate *in vitro* fosfomycin susceptibility testing, G6-P is recommended to be added to the culture medium (28). Our study elucidated further evidence for inconsistencies in bacteria resistance due to the culture medium. Although 7H10 agar is generally recommended as the standard medium for drug susceptibility tests (DSTs) for MTB (22), it can yield false-susceptible results for PAS susceptibility in strains with *metM* promoter mutations, potentially leading to incorrect clinical treatment decisions. For PAS, its recommended critical concentrations in 7H10 agar is 2 µg/mL (22). However, in the absence of methionine, the MICs of the *metM* promoter-mutated strains are only 0.125 µg/mL, the same as H37Ra, and are therefore not considered resistant. Consequently, our study suggests the use of 7H11 agar instead of 7H10 agar when testing the PAS susceptibility of the MTB strain.

In addition to spontaneous PAS resistance strain screening, substances antagonistic to PAS have long been applied to uncover the mechanism of PAS resistance (29, 30). The inhibitory action of PAS in MTB can be reversed by methionine, biotin, and certain fatty acids, amino acids, and purines, whereas the ability of methionine to reverse PAS resistance can be countered by other structurally related amino acids (29, 30). In the present study, the addition of methionine alone at a concentration approximately equal to that in human adult plasma (2–3 µg/mL) could not completely restore PAS resistance in strains with *metM* promoter mutations grown on 7H10 agar. Compared with 7H11 medium, 7H10 medium is primarily deficient in pancreatic digest of casein, leading to lower levels of biotin and probably certain fatty acids. Although the main antagonistic substance is methionine, we suspect that other active ingredients also play a certain role in antagonizing PAS, which could explain the differences in the resistance phenotypes of the *metM* promoter mutant strains in 7H10^MET^ and 7H11.

In a previous study, a collection of *in vitro* spontaneous PAS-resistant strains with three intergenic mutations located among *thyA-Rv2765*, *metM-Rv3254*, and *gplD2-lpdA* was documented (14). qPCR data revealed elevated *thyA*, *dfrA*, and *metM* expression, which potentially contributed to resistance to PAS (14). However, as mutations in *thyA* and *dfrA* are known to be related to PAS resistance, the relationship between *metM* promoter mutations and PAS resistance remained unexplained. In another study, disruption of MetM (BCG_3282 c) via transposon insertion resulted in a ≥30-fold decrease in methionine uptake in *M. bovi*s BCG, indicating that *metM* is the major facilitator of methionine transport. A *metM*-disrupted BCG strain exhibited a severe growth defect on Met-PAS plates and retained wild-type PAS susceptibility, indicating the involvement of *metM* in methionine-mediated PAS antagonism (31). This association is supported by the fact that *metM* sequences of MTB and *M. bovis* BCG are 100% identical. Not surprisingly, strains with *metM* induced expression due to the mutated 5′-end non-coding region or exogenous expression on plasmids can increase methionine uptake, thereby intensifying the antagonism of PAS and consequently increasing resistance. However, in our study, intracellular methionine levels significantly increased only in PAS-treated strains, suggesting that PAS might induce intracellular methionine deprivation, which is essential for biosynthesis and ultimately inhibits bacterial growth. To date, the basis of methionine-mediated PAS antagonism remains incompletely understood. While N-methyl-PAS remains effective against MTB, N,N-dimethyl-PAS has no anti-TB properties, suggesting that methionine inclusion may increase MTB’s ability to methylate PAS by elevating S-adenosylmethionine levels. This, in turn, may promote PAS inactivation via N,N-dimethylation by an unidentified methyltransferase (5). However, a recent study by Howe et al. discovered that the activated PAS form 2′-hydroxy-pteroate was effectively suppressed by methionine, indicating that the antagonistic effect of methionine on PAS is not solely attributed to N-methylation of PAS (31). Moreover, methionine-mediated antagonism required a functional PABA biosynthetic pathway in MTB, as the presence of methionine did not affect the susceptibility of the *pabB*-KO strain to PAS (31).

The accumulation of resistance-conferring mutations in the vicinity of the *Rv3253c*’s promoter region suggests the possibility of this region functioning as a transcriptional regulatory element. By referring to the transcripts of MTB in Interactive Genomics database (32), we identified the transcription start site (TSS) of *Rv3253c*, which is positioned 23 bases upstream from this gene (Fig S2). A putative promoter was identified on the −11 and −33 regions of the TSS predicted by the phiSITE PromoterHunter tool (33) (Fig S2). Since most mutations were clustered in this promoter region and its immediate flanking region, these mutations may alter the secondary structure, directly affecting the transcriptional efficiency of *metM*. Other mutations in more distant sites like C-101T could affect their interaction with potential repressive transcription factors and lead to improved transcription efficiency. While our experimental results have provided strong evidence that mutations in *metM* promoter can upregulate its expression, resulting in PAS resistance in MTB, further experiments are warranted to delve deeper into how these mutations upregulate the following gene expression.

It is noticed that the mutation sites of laboratory-induced isolates and clinical strains did not highly coincide. We found only two coincident mutations, C-42 and C-40, in both clinical and laboratory strains. Our understandings of the differences are as follows. Firstly, the incomplete phenotypic resistance data of clinical strains diminish the validation of the comparison between the diversity of resistant mutations of clinical isolates and laboratory strains. Secondly, clinical strains exist in diverse microenvironments, experience different treatment pressures, and encounter unique immune responses compared with laboratory-induced strains. These factors can greatly influence mutation selection and expression. Consequently, some mutations might be more readily identified in the laboratory, while real-world clinical complexities could suppress their occurrence. Conversely, certain mutations might prevail in clinical samples due to specific selection pressure. Thirdly, the genetic background and evolution time differ between the clinical strains and the laboratory isolates. Mutations are products of biological evolution and subject to dynamic changes over time. In our study, only an avirulent strain, H37Ra, was used to induce resistant isolates and these mutant isolates had been passaged only 1–2 times. However, the clinical mutant strains primarily belong to virulent MTB Lineage 2 or 4, which have evolved over an extended period. Therefore, *in vitro* laboratory screening, while a valuable tool to elucidate drug resistance mechanisms, identifies mutations influenced by experimental conditions and may not reflect long-term evolution. More data on clinical PAS drug-resistant strains are expected.

In summary, we have identified a new PAS resistance mechanism in MTB, involving mutations in the *metM* promoter region that result in increased *metM* transcription, increased intracellular methionine transport, and accumulation, ultimately leading to PAS resistance. Our findings provide new insights into the mechanism of PAS resistance in MTB, which could facilitate improved detection of PAS-resistant strains and enhancing treatment strategies.

## MATERIALS AND METHODS

### Isolation of spontaneous PAS-resistant mutants of MTB H37Ra

**Fig 9:**
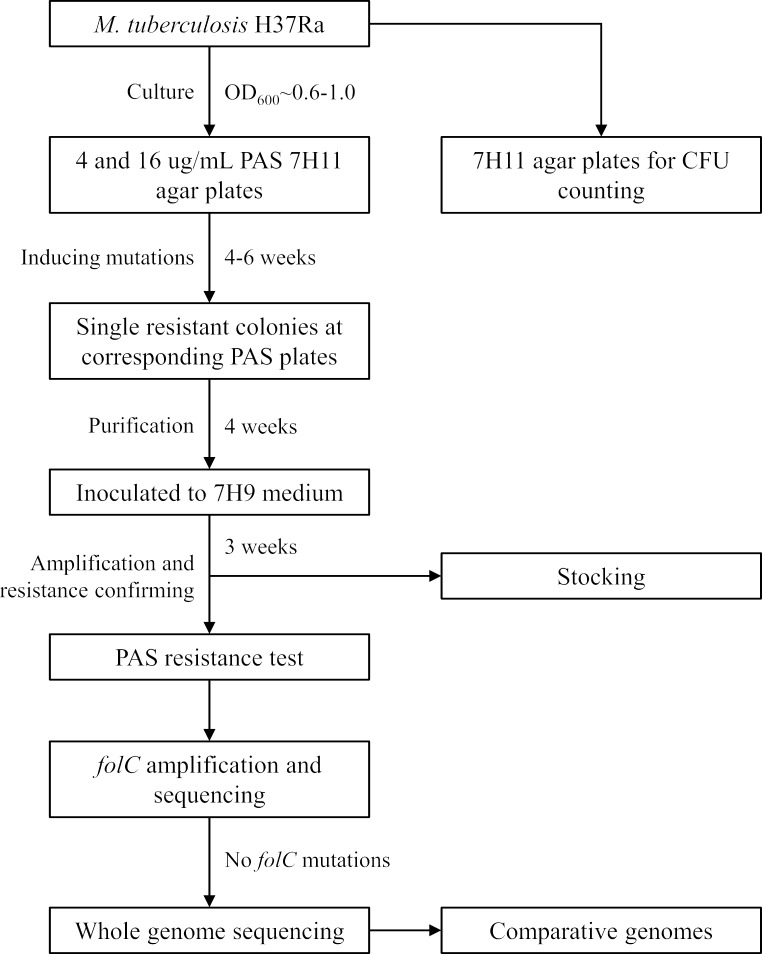
Workflow of the isolation and identification of spontaneous PAS-resistant mutants of MTB H37Ra.

MTB H37Ra (ATCC25177), generously provided by Prof. Honghai Wang from Fudan University (34), was initially inoculated and cultivated at 37°C in 7H9 broth (Difco, BD Biosciences, San Jose, CA, USA) supplemented with 10% ADC, 0.05% Tween-80, and 0.2% glycerol (7H9^ADC^) for 2–3 weeks to reach an optical density at 600 nm (OD_600_) of 0.6–1.0. ADC is an enrichment supplement recommended for the isolation and cultivation of mycobacteria, comprising 5% bovine serum albumin, 2% dextrose, 0.004% catalase, and 0.85% sodium chloride. Subsequently, the strain was streaked onto 7H11 agar plates (Difco, BD Biosciences) supplemented with 10% ADC and 0.5% glycerol (7H11^ADC^) with either 4 or 16 µg/mL PAS. These cultures were then gradient diluted onto 7H11^ADC^ agar for CFU counting. After 4–6 weeks of incubation, single colonies that grew on these PAS-containing plates were selected. To confirm the PAS resistance of these colonies, they were grown to the log phase in 7H9^ADC^ broth and then diluted to 0.1 McFarland standards, and then, 10 µL of bacteria suspension was inoculated into plates containing 7H11^ADC^ agar supplemented with a PAS concentration of 0, 0.0625, 0.125, 0.25, 0.5, 1, 2, 4, 8, 16, or 32 µg/mL. The plates were incubated at 37°C for 4 weeks. All PAS-resistant colonies were stocked and subjected to DNA extraction for further analysis (Fig. 9).

### Identification of mutant genes by WGS

Genomic DNA from all MTB strains was extracted using a DNeasy Blood & Tissue Kit (Qiagen, Hilden, Germany) following the manufacturer’s protocol. Because *folC* mutations frequently arise in spontaneous PAS-resistant mutants, *folC* mutants were identified via amplification and sequencing using the primers *folC*-F and *folC*-R (Table S3). The wild-type strain H37Ra and isolates harboring wild-type *folC* were subjected to WGS to identify novel mutants. Library construction and WGS of the isolates were performed as previously described (35). Single-nucleotide variants and insertions and deletions (ranging from 1 to 5 bp in size) in all PAS-resistant strains were sorted and called with more than 10 reads, using the MTB H37Ra genome (NC_0009525) as a reference. Mutations in proline–glutamic acid/proline–proline–glutamic acid family genes and regions with repetitive sequences were excluded from the analysis. Mutations in the parent strain H37Ra, compared with the genome available online (NC_0009525), were also excluded from the analysis.

### Isolation of total RNA and determination of gene expression using RT-qPCR

The relative expression levels of *metM* and *Rv3254* in each mutant were compared with those in H37Ra. MTB strains were inoculated at a ratio of 1:1,000 in 25 mL of 7H9^ADC^ broth, cultured to an OD_600_ of 0.8, and then harvested. The total RNA was extracted using a Bacterial RNA Kit (Omega Bio-Tek, Norcross, GA). cDNA was synthesized from the purified RNA (1,000 ng) using a PrimeScript RT Reagent Kit with gDNA Eraser (Takara, Kusatsu, Japan). RT-qPCR was performed using the corresponding primers for *metM* and *Rv3254*. The housekeeping gene *rpoA*, encoding the RNA polymerase α subunit, was employed for gene expression normalization. It was amplified using the primer pairs *rpoA*-rF and *rpoA*-rR in this study (Table S3) (36). The qPCR program consisted of a pre-incubation step at 95°C for 30 s, followed by 40 amplification cycles with denaturation at 95°C for 5 s and annealing at 60°C for 30 s. A melting curve analysis was performed to verify amplification specificity. PCR was repeated three times for *metM* and *Rv3254* and twice for *rpoA*. The 2^−ΔΔCt^ method was utilized for the relative quantification of gene expression, as described previously (37, 38).

### *metM* overexpression in MTB H37Ra

To initiate *metM* overexpression, *metM* along with its 300-bp 5′-end non-coding sequence (wild-type promoter) from H37Ra and the mutant P39 carrying the C-57T mutation in its promoter were amplified using specific primers (Table S3) and Q5 High-Fidelity DNA Polymerase (NEB, USA). The amplification protocol consisted of initial denaturation at 98°C for 2 min, followed by 35 cycles of denaturation at 98°C for 15 s, annealing at 62°C for 15 s, and extension at 72°C for 1 min. Subsequently, the PCR products were purified using a QIAquick PCR Purification Kit (Qiagen), digested with the enzymes *Xba*I and *Acl*I (NEB), and ligated to the shuttle vector pOLYG (39). The pOLYG vector was also digested with the same enzymes before ligation to yield recombinant plasmids. These recombinant constructs were then transformed into H37Ra, as described previously, to yield the *metM* overexpression strains Ra::*metM*OE and Ra::C-57Tpro-*metM* (Table S4) (40).

An alternative effective promoter, namely, the *lysG* promoter (41), was also used to construct the overexpression strain Ra::*lysG*pro-*metM*. Furthermore, because MetM in *M. smegmatis* was found to share 73% identity with MTB at the amino acid level, we constructed a recombinant plasmid using the *lysG* promoter and *metM* of *M. smegmatis* (*MS_metM*). Subsequently, this plasmid was transformed into H37Ra to generate Ra::*lysG*pro-*MSmetM*. We then examined whether this construct had a similar effect on PAS resistance. Briefly, the 300-bp 5′-end non-coding sequence along with an 18-bp coding sequence of *lysG*, from H37Ra, was amplified to serve as the *lysG* promoter. The entire *metM* sequence was amplified from H37Ra and *M. smegmatis* mc^2^155. Each fragment was independently PCR amplified, and then, the *lysG* promoter and *metM* were combined by overlap PCR. The PCR conditions were consistent with those mentioned earlier, with variations in annealing temperature and extension time, as detailed in the Supplementary Data, along with all primers used (Table S3). Subsequent steps, including purification, restriction digestion, ligation, and transformation, were performed as previously described to generate the overexpression strains Ra::*lysG*pro-*metM* and Ra::*lysG*pro-MS*metM* (Table S4). To validate *metM* and *MSmetM* overexpression, RT-qPCR was performed using the respective primers for *metM* in strains Ra::*metM*OE, Ra::C-57Tpro-*metM*, Ra::*lysG*pro-*metM*, and *MSmetM* in strain Ra::*lysG*pro-*MSmetM* and the aforementioned methods. Ra::pOLYG served as a vector control, and the relative expression levels were compared with those of H37Ra.

### Determination of promoter activity of intergenic region mutations using the *lacZ* reporter vector

To assess the impact and direction of intergenic region mutations, a fusion reporter vector containing the intergenic sequence between *metM* and *Rv3254*, with *lacZ* as the reporter gene, was constructed. The mutants P14, P41, and P120, as well as the wild-type MTB strain H37Ra, served as templates for obtaining the corresponding promoter sequences. Briefly, the 300-bp 5′-end non-coding sequence of either *metM* or *Rv3254* as the promoter was amplified by PCR using specific primers. After purification, the fragments were digested with the enzymes *Xba*I and *Bam*HI (NEB) and ligated to the plasmid placZ, which had been digested with the same enzymes, to generate recombinant plasmids. The placZ vector, a non-integrating vector equipped with a kanamycin resistance cassette, was kindly provided by Dr. Peng Cui, and its structure schematic is presented in the Supplementary Data (Fig S3). Four *metM* recombinant plasmids and three *Rv3254* recombinant plasmids were constructed (Table S4). These recombinant plasmids were subsequently transformed into *M. smegmatis* mc^2^155.

The intracellular β-galactosidase activity of the transformed *M. smegmatis* strains was quantitatively analyzed using a β-galactosidase assay kit (Invitrogen, Thermo Fisher Scientific, Waltham, MA, USA). The procedures were based on the manufacturer’s instructions with slight modifications. Briefly, sample preparation was conducted according to standard procedures to obtain the cell lysate. Beta-galactosidase experiments were performed in a 96-well format with three replicates. Throughout the reaction, the plate remained stationary, and OD_420_ was continuously measured using the SpectraMax Paradigm Microplate Reader (Molecular Devices, USA). The reader was maintained at 37°C with the “Kinetic” read type selected, running for a total of 30 min, with 5-min intervals for continuous observation. Then, linear curve fitting was performed using GraphPad Prism 9.0.0 (GraphPad, San Diego, CA, USA). An acceptable linear regression coefficient of correlation was considered to be 0.9 or higher; otherwise, re-experimentation was required. The β-galactosidase enzyme reaction rate was determined as the slope of the curve (*K*). The specific activity of the lysate was calculated using the formula: specific activity = *K*/mg protein. Protein quantification was conducted using the BCA assay (Pierce Chemical, Thermo Fisher Scientific) according to the manufacturer’s instructions. Statistics were carried out using GraphPad Prism 9.0.0 (GraphPad, San Diego, CA, USA) software.

### Drug susceptibility tests

DSTs were performed for all nine PAS-resistant mutant strains and the parent H37Ra strain on 7H11^ADC^ or 7H10 agar (Difco, BD Biosciences) plates supplemented with 10% ADC and 0.5% glycerol (7H10^ADC^). To assess the susceptibility of *metM*-overexpressing strains to PAS, DSTs on Ra::*metM*OE, Ra::C-57Tpro-*metM*, Ra::*lysG*pro-*metM*, and Ra::*lysG*pro-*MSmetM* were performed on 7H11^ADC^ agar containing varying concentrations of PAS (0.0625, 0.125, 0.25, 0.5, 1, 2, 4, 8, 16, and 32 µg/mL), in which the parent strain H37Ra, nine mutants, Ra::pOLYG, Ra::*metM*OE, Ra::C-57Tpro-*metM,* and vector control Ra::pOLYG were conducted in same batch. Each strain was inoculated at an approximate inoculum size of 3 × 10^7^ CFU/mL × 10 µL, and triplicate assays were conducted.

To investigate the effect of methionine (0, 2, 3, 4, 6.3, or 20 µg/mL) on PAS susceptibility on a 7H10 plate, the mutants P14, P39, P41, and P120; *metM*-overexpressing strains Ra::*metM*OE and Ra::C-57Tpro-*metM*; vector control Ra::pOLYG; and wild-type strain H37Ra were selected. Valine and tyrosine of 20 µg/mL served as controls. Each strain was inoculated on the plates in duplicate, and the whole assays were repeated twice.

### Free amino acid concentration determination

Amino acid concentrations were determined following a previously described method (42). In brief, cultures were thoroughly mixed with 1.0 mL of ice-cold methanol/acetonitrile/H_2_O (2:2:1, vol/vol/vol). The resulting homogenate was sonicated in an ice bath for 30 min for protein deposition and then centrifuged for 20 min (14,000 × *g*, 4°C). The supernatant was subjected to analysis using an UHPLC system (1290 Infinity LC, Agilent Technologies, Santa, Clara, CA, USA) coupled to a QTRAP system (AB Sciex 5500, Sciex, Framingham, MA, USA). Then, the chromatographic peak area and retention time of the extract were determined using Multiquant software (Sciex), and the retention time was corrected using standard amino acids and their derivatives to identify metabolites. One quality control sample was created for every eight experimental samples in the sample cohort to test and evaluate the stability and repeatability of the system. All standard amino acids and their derivatives, as well as isotope standard materials, were purchased from Sigma-Aldrich (St. Louis, MO, USA). The metabolites detected in four or six samples with coefficients of variation smaller than 30% were denoted as reproducible measurements. Statistics were performed using GraphPad Prism 9.0.0 (GraphPad, San Diego, CA, USA) software.

### Growth curve

To investigate whether mutations in the *metM* promoter resulted in growth defects, the growth rate of nine strains with *metM* promoter mutations were compared with that of H37Ra. In brief, H37Ra and mutant strains cultured in 7H9^ADC^ broth were diluted in fresh medium to a 1.0 McFarland standard, inoculated in 5 mL of 7H9^ADC^ broth at a 1:100 ratio, and then incubated at 37°C. Culture samples (200 µL) were taken for bacterial density measurements at OD_600_ using a microplate reader in the 96-well format with three replicates. Measurement was performed every 3 days until day 12 and every 2 days thereafter. Statistical significance was calculated using two-tailed Student’s *t*-test, with *P* < 0.05 indicating statistical significance.

## SUPPLEMENTAL MATERIAL

Fig. S1 mbio.02073-23-s0001.pdf Correlation of OD_420_ and time of β-galactosidase assays in 30 min.

Fig. S2 mbio.02073-23-s0002.pdf Schematic diagram of *metM* promoter region.

Fig. S3 mbio.02073-23-s0003.pdf Structure sketch map of placZ.

Table S1 mbio.02073-23-s0004.xlsx Quantitative analysis of amino acids and their derivatives by MRM.

Table S2 mbio.02073-23-s0005.docx Mutations in the *metM* promoter region in clinical MTB strains.

Table S3 mbio.02073-23-s0006.xlsx Primers used in this study.

Table S4 mbio.02073-23-s0007.docx Bacterial strains and plasmids constructed in this study.

ASM does not own the copyrights to Supplemental Material that may be linked to, or accessed through, an article. The authors have granted ASM a non-exclusive, world-wide license to publish the Supplemental Material files. Please contact the corresponding author directly for reuse.
