# Supplementary material for: Mutations in the promoter region of methionine transporter gene metM (Rv3253c) confer para-aminosalicylic acid (PAS) resistance in Mycobacterium tuberculosis
Source: mBio. 2024 Jan 5;15(2):e02073-23. doi: 10.1128/mbio.02073-23 (PMC10865796; doi:10.1128/mbio.02073-23)
Supplement: Fig. S1 — Correlation of OD420 and time of β-galactosidase assays in 30 min. [file mbio.02073-23-s0001.pdf]

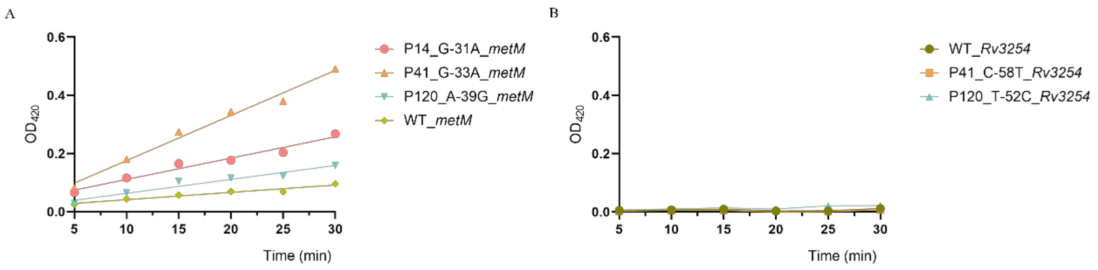

S1 Fig. Correlation of OD_{420} and time of β-galactosidase assays in 30 min. (A) For *metM* promoter, the OD_{420} of the reaction systems was linearly related to the reaction time no matter wild-type or mutants. (B) For *Rv3254* promoter, the value of OD_{420} showed no obvious change over time during the reaction.
