# Supplementary material for: Mutations in the promoter region of methionine transporter gene metM (Rv3253c) confer para-aminosalicylic acid (PAS) resistance in Mycobacterium tuberculosis
Source: mBio. 2024 Jan 5;15(2):e02073-23. doi: 10.1128/mbio.02073-23 (PMC10865796; doi:10.1128/mbio.02073-23)
Supplement: Fig. S2 — Schematic diagram of metM promoter region. [file mbio.02073-23-s0002.pdf]

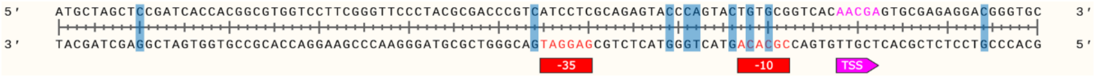

S2 Fig. Schematic diagram of *metM* promoter region. Magenta arrow marks the transcription start site (TSS). Red boxes indicate possible core promoter, including -10 and -35 box. Blue transparent boxes represent mutations conferring PAS resistance screened in our study.
