# Supplementary figures and images for: Mutations in the promoter region of methionine transporter gene metM (Rv3253c) confer para-aminosalicylic acid (PAS) resistance in Mycobacterium tuberculosis
Source: mBio. 2024 Jan 5;15(2):e02073-23. doi: 10.1128/mbio.02073-23 (PMC10865796; doi:10.1128/mbio.02073-23)

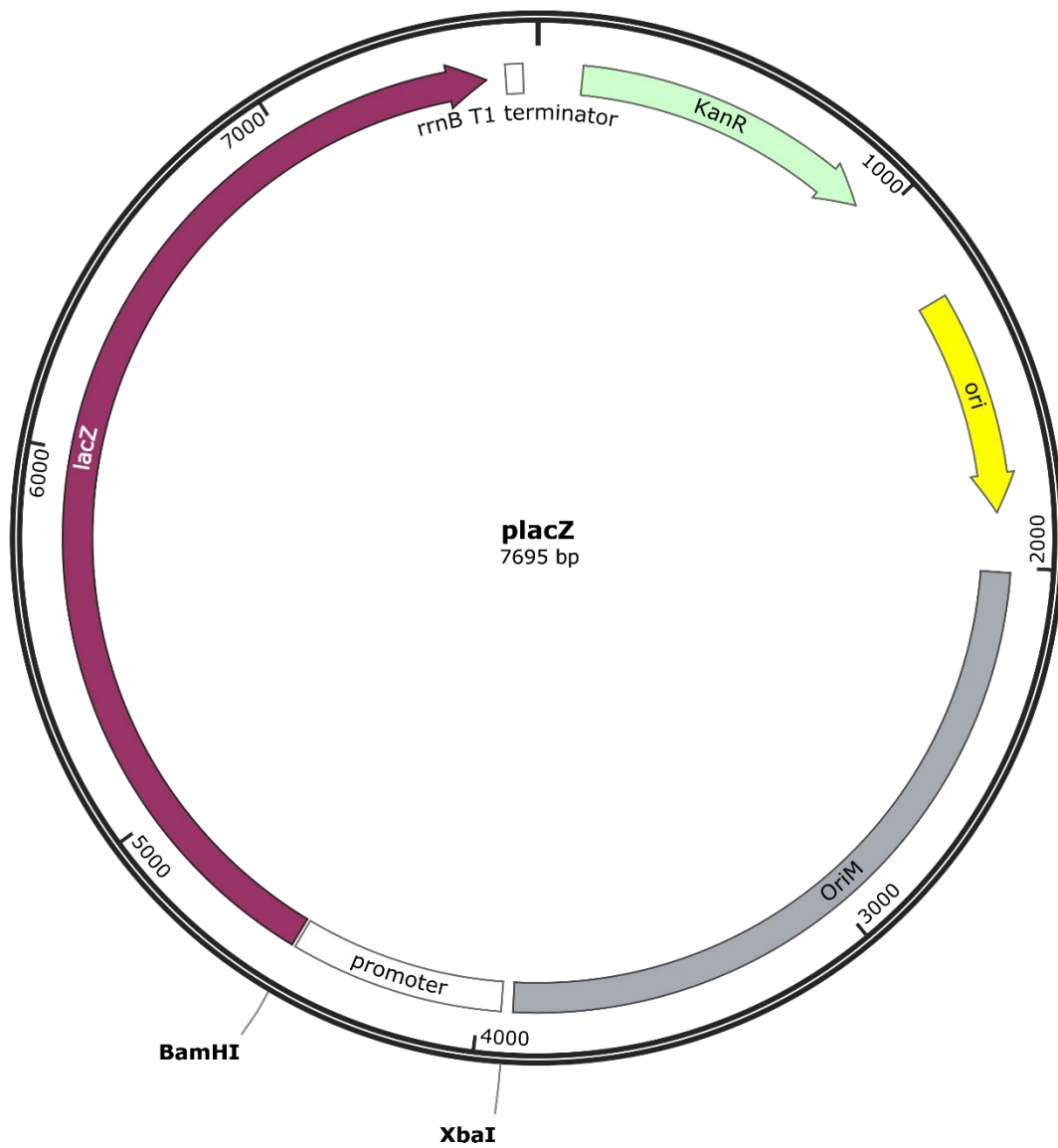

**S2 Fig.** Structure sketch map of **placZ**.

Supplement: Fig. S3 — Structure sketch map of placZ. [file mbio.02073-23-s0003.pdf]
