# Supplementary material for: Mutations in the promoter region of methionine transporter gene metM (Rv3253c) confer para-aminosalicylic acid (PAS) resistance in Mycobacterium tuberculosis
Source: mBio. 2024 Jan 5;15(2):e02073-23. doi: 10.1128/mbio.02073-23 (PMC10865796; doi:10.1128/mbio.02073-23)
Supplement: Table S2 — Mutations in the metM promoter region in clinical MTB strains. [file mbio.02073-23-s0005.docx]

**Table S2. Mutations in the *metM* promoter region in clinical MTB strains**

| Mutation | Strain accession number |
| --- | --- |
| A-19G | SRR6153046 |
| C-21T | ERR2513837 SRR6044946 SRR6045284 SRR6045400 SRR6045419 SRR6045557 |
| C-40G | ERR2514877 |
| C-41T | ERR2513239 |
| C-42T | UM200902T0099 |
| A-46C | ERR2513241 ERR2516842 ERR2516853 ERR2516886 ERR2516898 ERR2517046 |
| A-48G | SRR6153161 |
| T-58C | ERR117451 |
| G-59C | ERR2513898 ERR2516755 ERR2516860 |
| A-63G | ERR2516515 |
| G-75T | ERR2041683 ERR2041686 ERR2041688 ERR2041689 ERR2041698 ERR2041699 ERR2041709 ERR2041712 ERR2041718 ERR2041723 ERR2041731 ERR2041734 ERR2041736 ERR2041737 ERR2041738 ERR2041739 ERR2041746 ERR2041748 ERR2041749 ERR2041753 ERR2041754 ERR2041756 ERR2041759 ERR2041762 ERR2041763 ERR2041764 ERR2041766 ERR2041777 ERR2041780 ERR2041785 ERR2041786 ERR2041789 ERR2041790 ERR2041791 ERR2041795 ERR2041797 ERR2041798 ERR2199914 ERR2200093 |
| C-78T | ERR2514657 UM200902T0079 UM201228T0025 |
