# Supplementary material for: Mutations in the promoter region of methionine transporter gene metM (Rv3253c) confer para-aminosalicylic acid (PAS) resistance in Mycobacterium tuberculosis
Source: mBio. 2024 Jan 5;15(2):e02073-23. doi: 10.1128/mbio.02073-23 (PMC10865796; doi:10.1128/mbio.02073-23)
Supplement: Table S4 — Bacterial strains and plasmids constructed in this study. [file mbio.02073-23-s0007.docx]

**Table S4.** **Bacterial strains and plasmids constructed in this study**

| Strain Name | Recombinant plasmid | Recombinant genes | Description |
| --- | --- | --- | --- |
| Ra::*metM*OE | pOLYG | Wild-type *metM* promoter and *metM* of MTB H37Ra | H37Ra-containing plasmid pOLYG-*metM* |
| Ra::C-57Tpro-*metM* | pOLYG | Mutant *metM* promoter (C-57T) and *metM* of MTB H37Ra | H37Ra-containing plasmid pOLYG-C-57Tpro-*metM* |
| Ra::*lysG*pro-*metM* | pOLYG | *lysG* promoter and *metM* of MTB H37Ra | H37Ra-containing plasmid pOLYG-*lysG*pro-*metM* |
| Ra::*lysG*pro-*MSmetM* | pOLYG | *lysG* promoter and *metM* of *M. smegmatis* | H37Ra-containing plasmid pOLYG-*lysG*pro-*MSmetM* |
| *MS*::placZ-P14_G-31A_*metM* | placZ | Mutant *metM* promoter (G-31A) and *lacZ* of *E. coli* | *M. smegmatis* mc^2^155*-*containing plasmid placZ-P14_G-31A_*metM* |
| *MS*::placZ-P41_G-33A_*metM* | placZ | Mutant *metM* promoter (G-33A) and *lacZ* of *E. coli* | *M. smegmatis* mc^2^155-containing plasmid placZ-P41_G-33A_*metM* |
| *MS*::placZ-P120_A-39G_*metM* | placZ | Mutant *metM* promoter (A-39G) and *lacZ* of *E. coli* | *M. smegmatis* mc^2^155-containing plasmid placZ-P120_A-39G_*metM* |
| *MS*::placZ-WT_*metM* | placZ | Wild-type promoter of *metM* and *lacZ* of *E. coli* | *M. smegmatis* mc^2^155-containing plasmid placZ-WT_*metM* |
| *MS*::placZ-P41_C-58T_*Rv3254* | placZ | Mutant *Rv3254* promoter (C-58T) and *lacZ* of *E. coli* | *M. smegmatis* mc^2^155-containing plasmid placZ-P41_C-58T_*Rv3254* |
| *MS*::placZ-P120_T-52C_*Rv3254* | placZ | Mutant *Rv3254* promoter (T-52C) and *lacZ* of *E. coli* | *M. smegmatis* mc^2^155-containing plasmid placZ-P120_T-52C_*Rv3254* |
| *MS*::placZ-WT_*Rv3254* | placZ | Wild-type *Rv3254* promoter and *lacZ* of *E. coli* | *M. smegmatis* mc^2^155-containing plasmid placZ-WT_*Rv3254* |
